# Orientalism

**Published:** 1861-10

**Authors:** 


### Art. V.—ORIENTALISM.

During the Middle Ages, intercourse between Europe and Asia was almost entirely suspended. Far from seeking to unite, each was, by the course of events, separated more and more from the other. There was nothing in common between the asceticism of the cloister and the soft affluence of the curtained verandah. For a moment, indeed, they may have interchanged a few tokens of recognition, for it is recorded of Charlemagne that, in the eighth century, he opened some diplomatic relations with Harun al Reshid,* our early friend of the *Arabian Nights' Entertainments;* but with the exception of this transient glimpse, Asia remained hermetically sealed against Europe for the space of nine long and gloomy centuries.† Even the Crusaders sought nothing beyond Jerusalem delivered and the seaboard of the Holy Land. In the estimation of those high-minded soldiers and devotees, the depths of Asia were the regions of heathenism, sorcery, and spiritual darkness. In the charming cadence of his well-regu-

---

* Gibbon, c. 49, vol. v. 4to ed., p. 145.

† Some distant communication was always kept up by means of commerce. During the Middle Ages, Antioch, Damascus, and Aleppo carried on the East India trade with Constantinople, Venice, and Genoa; but these cities sank into insignificance when trade took another direction, and went round by the Cape.

lated stanzas, Tasso has represented the prevailing notions of mankind in this respect, not merely with the eye of a poet, but with the pen of a master. About two hundred years before the discovery of America by Columbus, Marco Polo had traversed some portions of the East; and the mystery of the Indies was at last cleared up by the rounding of the Cape of Good Hope. The European family congratulated itself on discovering its own cradle in the East, and started anew on the laudable task of exploring the entire circle of the globe. To Sir William Jones may be ascribed the original merit of making us first acquainted with the language and poetry of the Hindoos, in the same manner as Sir Henry Rawlinson and Mr. Layard have, some fifty years later, restored to us the long-lost arcana of Persia and the monumental archives of Babylon.

The influence of Orientalism has been gradually on the increase throughout Europe for the last three hundred years. Camoens, who, as far as we can understand him, is but a sorry writer, owes what interest he possesses to a tropical subject, mixed up with a strange confusion of pagan gods and godesses, grossly out of place between the mouth of the Tagus and Trincomalee. In this country, the Oriental idea dates back as far as a hundred years or more. Collins' *Oriental Eclogues,* which are masterpieces of their kind, particularly the second eclogue, were composed about a century ago, 1740. *Rasselas* is Oriental: the *mise en scène* is entirely Oriental: the sentiments are gloomy, without a ray of hope, and imbued with a dark tinge of fatalism. *Vathek,* the brightest of dreams and the most eloquent of tales, the most panoramic of scenes, and the most densely populated of novels, is unmitigated Orientalism. *Lalla Rookh* is the same—soft, effeminate, showy, and evanescent. None of these works of fiction, not even excepting *Rasselas,* the first among the first-born of sturdy writers, pretends to satire, or to that acute dissection of society which is the property of Pope and Molière among the moderns, or Terence and Plautus among the ancients. On the contrary, what they cannot approve of they lament, and what they cannot correct they disdain. But such is the relish for Orientalism among modern readers, that the costume alone is sufficient to give them a passport to a good reception. The sight of the turban is enough. It was so of late. The interest awakened by the Crimean war touched a sensitive chord in every nation of Christendom, which responded to the call, and hastened to the aid of the Turk threatened by the overpowering forces of Romanoff. There must be some universal feeling alive in the present day, that finds its analogue in the heart of the East, although it is not easy to say in what that analogy consists. For the morals of Mahometanism are not, nor ought to be, the same as ours. They are not those of ancient Greece or Rome at their most depraved periods. The stern censorship of Tacitus or Juvenal, and the keen irony of Horace, have no counterpart in anything we read of in the history of the Osmanli Turks.* Pagan society never reduced woman to the infamy of the harem or the seraglio.† Polygamy was never in vogue among them, nor even so much as countenanced by the Cæsars of Italy or the demigods of Greece. Their morality was low enough, but most assuredly it was exempt from this disgrace, at least. Possibly, Mahomet never intended to introduce the practice, but was carried away by the pressure of the times, and forced to yield to the popular inclination; and it was far easier to put the cimeter in the hands of his followers, and bid them go forth and conquer, than to prescribe for them rules of virtue which would never be observed. Sanctity and chastity were beyond his reach. Wherein, then, does the sympathy between the East and the West consist? Is it in the softness of social manners, and those luxurious modes of life that foster sensuality? or in a style of literature morbidly sensitive and overre-fined? or in the love of wealth and parade? or in none of these, but only in a passing fancy of the age? And yet one would think it is something deeper than this.

Look at the First Napoleon—his character is scarcely occidental. Its wildness, its swooping genius belongs to the East. It reminds us of Tamerlane or Zenghis Khan, or those Saracenic chiefs who swept along Africa, crossed the Straits of Gibraltar,

---

* Creasy's *Ottoman Turks*, 2 vols. Bentley, 1856.

† THE ROYAL MARRIAGE LAW OF TURKEY.

*To the Editor of the Times.*

Sir,—Permit me to correct an error in your article on the state of Turkey. You speak there of "four or five wives of Abdul Medjid." But it is matter of history that no Sultan of the Ottoman race has been legally married since the days of Bajazet the Great. On his capture by Timur, after the battle of Angora, the Sultana was treated with gross insult, and to guard against the shadow of a chance of such a disgrace recurring no inmate of the Seraglio has for more than 400 years been a legitimate wife according to Mussulman law.

July 13, 1861. CANTEMIR.

"When one considers that the most beautiful girls among the Mussulman population of the empire usually find their way to the Bosphorus, and that, besides these, Circassia and Georgia contribute largely to the harems of Constantinople, the number of really pretty women to be seen at the Sweet Waters or other places of resort appears small."—*Times*, July 22, 1861.

There were in the worst times among the Gentiles, some few in whom, as Cicero says in his defence of Sextus Roscius, Amer. X., alluding to Cæcilia Metcho, the wife of Sylla, remained, as if for the sake of example, the vestiges of ancient duty—in quâ muliere, quasi exempli causâ, vestigia antiqui officii remanent. And Pliny speaks in remarkable terms of Fannia, a Roman matron, 'quæ sanctitas! quanta gravitas! quanta Constantia! non minus amabilis quam veneranda!"—*Epist.* vii. 19. The language could not be more devout in the brief for the canonization of some saintly virgin or widow. Tacitus mentions Occia, a vestal virgin, as a woman *summæ sanctimoniæ.* And yet Cicero divorced himself from Terentia, to whom he had been married for thirty years, and Cæsar repudiated his exemplary spouse because she had been unjustly aspersed.

occupied Spain, penetrated the passes of the Pyrenees, and broke their strength against the steel-clad chivalry of the North. So strong is the taint of Orientalism, or of Saracenic blood, in Napoleon, that he was usually followed into battle by a mounted Arab in true Asiatic costume. His proclamations, which have been so severely criticised and censured, breathe the spirit of Oriental poesy in the court and camp. When he shouted to his soldiers, *Vous étes descendus des Alpes comme un torrent,* or when, on another occasion, he declared, *Je suis le dieu des armées,* did he use the polite and courtly phraseology of Louis XIV. ? Was it not rather the language of Eastern hyperbole, such as might have been spoken by the Mahomet of the West ? At Aboukir, Cairo, or Mount Tabor, Napoleon rehearsed or found a language of his own.

His life is an epic belonging to the class of heroic legends. He touched the four quarters of the globe. He disputed our arms in India and America at the same time that he bestrode the confines of Asia, Europe, and Africa with the armed tread of a warrior. And yet, the same hand that hewed the Simplon and carried the flag across the bridge of Lodi, was the first to accept from the pen of St. Bernardin de St. Pierre the gentle dramatic tale of Paul and Virginia. He is always poetic and sublime. On the top of the eternal pyramids or amid the flames of the burning Kremlin, at the glorious sunrise on the plains of Austerlitz, or upon the solitary rock amid the waves of the wide Atlantic, he stands single and alone. He is the chief object of interest, when, in the zenith of his power, he dictated the terms of peace and war from his imperial throne at the Tuileries, or, when in the day of his reverse, he turned pale in the gloomy grandeur of defeat on the field of Waterloo. He ruled the spirit of the age, and impressed on the destiny of mankind an image of his own, as ineffaceable as the hand of time, and as enigmatical as that of a genie of the East.

The next potentate over the minds of men was the poet Byron. No one, merely by the aid of his pen, ever effected so great a change in thought and sentiment as the author of *Childe Harold.* In the writings of the *Poets of the Lakes,* as they were styled, as well as in those of the pantheist Shelley, whose fancy seems to have followed some Indian ideal, it would be easy to point out the Oriental spirit that actuates them all. The reader will call to mind Coleridge's *Khubla Khan,* and Southey's *Last of the Goths.* Even the charm that pervades Sir Walter Scott's exquisite lyrics is due to their Oriental character, which is derived from the Crusades, and mixed up with a thousand mystic myths and mediæval reminiscences. But Byron surpasses them all in breadth of colouring and grasp of intellect, and includes within his glowing themes the burden of the rest. He once thought of visiting India, and in fact made some preparations for the journey,* but this project was subsequently restricted to a tour through the Morea, and a sojourn at Constantinople, in company with his friend Hobhouse. A new tie was thus formed between the spirits of the East and West, each link of which was forged of gold or diamonds by the touch of Byron. He alone related for the first time as a matter of fact, what Goethe had chanted only as a fond idea,

> Know ye the land where the cypress and myrtle
> Are emblems of deeds that are done in their clime?

And he alone in *Childe Harold's Pilgrimage* lighted up with the fire of a true poet those scenes of nature which captivate the senses and exalt the soul.† But *Childe Harold* revels chiefly on the shores of the Levant, and sinks into luscious repose amid the ruins of ancient Greece, beneath the azure sky that suffuses the waves and mountains of the East. The contrast is strikingly drawn between European activity and Oriental apathy and inaction. The remains of Athens, Troy, and Corinth bask in the sunshine, or slumber amid roses and olives. The silence of the scene is broken only by the spirit of the West. "Lara," the "Corsair," the "Giaour," "Mazeppa," and the "Bride of Abydos," are Europeans in Asiatic costume. The British temper breathes, and pants, and burns, and energizes beneath the truban, the pelisse, the Oriental trouser, the pointed slipper, and the naked cimeter. As actors they play their parts to perfection, but it is not natural to them. They smack of the sands of Araby the Blest; but their speech betrays them, and they talk English. Even the haughty feudal baron, Manfred the Misanthrope, who converses with the snow-clad peaks of Switzerland, forgets himself, and invokes Ahriman and Ormuzd, the dæmons of the East. The fire-fays and elves of Persia appear upon the crags and vanish in the mists that boil up about the glaciers of the Wengern Alp. The fiction is consummately worked out; but the personages do not properly belong to the eastern or western hemisphere. To what particular country do Gulnarez, Medora, Kaled, Zuleika, or Leila belong? Certainly not either to England or Turkey. They are fatalists inspired with the impulsive temper of the North. Medora, the dreamy, pensive, brokenhearted Medora, is not a genuine Algerine. She is a Scotch lassie at heart, and a pretty little Christian playing the Turk.‡

---

* Moore's *Life of Byron*. Murray. 1860. p. 73.

† These scenes are now consigned to those useful pages, bound in a limp red cover, and 'ycleped *Murray's Handbook for Travellers*.

‡ The *furor* that still actuates the public in favour of Byron may be exemplified by the following paragraph:—

Sale at Newstead Abbey (Notts).—On Friday there was a sale by auction

But it is in Germany that the Oriental spirit has made the greatest progress. It is not possible to say when, where, nor how it first entered and mixed itself up with the German tongue, whose Indo-Germanic root is now universally acknowledged. The speculative turn of thought, or idealism, of their philosophy reminds us of the Gymnosophists of Hindoo. The ancient German mythology is almost entirely Eastern and extravagant. Odin's skull is a microcosm comprehending the universe. Its present analogue is the pantheism of the German schools.

Goethe embellished his poems with the choicest Asiatic morsels and allusions. Sometimes it is an Indian legend which becomes an ode to the Deity—a pearl, as it were, from the gulf of Golconda finely engraved by the lapidary from Weimar: at other times he breathes nothing but Islamism. Collected as if in Divan, his couplets seem to have been copied from the dome of the mosque at Mecca. The style of thought, sentiment, and language is entirely Asiatic—not Asiatic and European, the Cross and the Crescent combined—but pure and absolute Orientalism—oppressively calm and superbly monotonous—the coloured daylight of the Alhambra, the twilight of the East. Lara, the Giaour, and the Corsair find no footing there.

Closely associated with this Oriental turn of thought is a profound melancholy and an air of inveterate scepticism. It has been a fashion of late years to feign this mournful tone, perhaps in imitation of its distinguished prototype, Byron.* It is sup-

---

at the Abbey of valuable effects, formerly the property of Lord Byron. Many of the lots realized only moderate prices. Four papier-maché decanter stands, formerly Lord Byron's, were sold at 15*s.*, the positive value of all being about 4*d.* The model of a frigate, the property of the deceased poet, sold at 3*l.* 15*s.*, the value being less than half that sum. A snuff-box, with a portrait of Byron, but not stated to have any other association, went at 10*s.* A parian figure of a sleeping Cupid, the property of Lord Byron, sold at 15*s.* The first printed copy of his early poems, with autograph, after a vigorous competition, fetched only 6*l.*, Mr. Webb being the purchaser; and a pair of brass candlesticks, used by his lordship in college, were bought in by the same gentleman at 3*l.* 10*s.* Lord Byron's punch-bowl, broken, but repaired, and not, perhaps, worth one shilling, realized 3*l.* 5*s.* A marble bust, life size, of Charles I., on marble half column, chiselled with great delicacy, sold at 15*l.*, to Mr. Redfern, of Warwick; and a bust, in the same size and style, of William III., at the same price, to Mr. Woodgate, of London. Musical instruments and portfolios of music, the former embracing flutes, guitars, harpsichords, musical boxes, and harps, and the latter copies of the best operas and standard classical music, brought good prices. There were pipes of every design and pattern, in which cost became paramount to utility, and in all cases the articles put up were disposed of at fabulous prices. Some curious and valuable articles in Dresden china, plate, and plated goods were subsequently disposed of to advantage, and the sale concluded with the wine department, some small lots of hock of 1818, a few dozens of the same wine "from Lord Byron's cellar," and a large quantity of various wines of later vintages, having gone off at large premiums.

* Moore mentions the disposition to melancholy which belonged to his temperament—the boundary of this world's pleasures—"to see nothing but 'clouds and darkness' beyond, was the doom, the anomalous doom, which a nature premature in all its passions and powers inflicted on Lord Byron."—*Op. cit.* p. 87.

posed to exhibit something aristocratic in its look and bearing; but nothing can be in worse taste—it is simply a vulgarity, if feigned, or a constitutional ailment, if genuine. At the best, it is nothing better than pyrrhonism, or universal doubt, such as that cultivated by Lucian, Lucretius, or Voltaire. It doubts everything, except the right of doubting; it almost doubts of its own being; and rushes headlong into pantheism to escape from unqualified Atheism. It philosophizes on this world and the next; and perceiving that it is impossible to uproot Christianity, it perplexes itself with lopping its boughs and twisting its twigs into every variety of shape, to suit the whim or fancy of the hour. No one dares face an eternity of nothingness. The vast void must be filled up with fiction or truth—truth with a day of judgment, or fiction without a hope.

From this dread abyss uprose Faust. Having nothing in common with the transcendental philosophy of Lucian, Montaigne, or Voltaire, Faust is an absolute sceptic. His thirst is universal knowledge—his drink an intoxicating spirituality. He ransacks nature, art, science, passion, the city, and the desert, to get at the grand arcanum of existence. He is not an alchymist foiled in transmuting a grain of dust into an atom of gold; he is much more than this, for he aims at bringing down the Almighty into his crucible. The end is Margaret, dishonour, grief, and woe; and in the grand finale, the scene darkens, hell yawns, and the curtain drops over a chasm of eternal night. Such is the climax of Orientalism in the persons of Faust and Mephistopheles, a twofold blasphemy that shivers to nothing the natural and supernatural order of events.

Nor is English literature free from the blight of this dismal mental obliquity. In one of the best stanzas of his *Elegy in the Churchyard*, Gray thus soliloquizes :—

> "For who to dumb forgetfulness a prey,
> This pleasing, anxious being e'er resign'd,
> Left the warm precincts of the cheerful day,
> Nor cast one longing, ling'ring look behind?"

It is evident these lines were paraphrased from the far nobler ones in *Paradise Lost*:—

> "———— for who would lose,
> Tho' full of pain, this intellectual being,
> Those thoughts that wander thro' eternity,
> To perish, rather swallow'd up and lost,
> In the wide womb of uncreated night,
> Devoid of sense and motion?"

It is true that these sentiments are put into the mouth of one of the fallen angels, but the relish with which they are quoted, as they stand alone, betrays the kindred feeling with which they are read. In another stanza of his *Elegy*, Gray says:—

> "Each in his narrow cell for ever laid,
> The rude forefathers of the hamlet sleep."

The words *sleep* and *for ever* may be excused on the plea of poetic licence, but taken as they are, the expression is unqualified, and they mean precisely what they say. There is more real piety and hope of futurity in many an ode of Horace than in this; Virgil, in his sixth *Ænead*, allows of no sleep among the dead, either for good or evil; nor does Dante in his *Divina Commedia*. With each of these two last-named poets everything is in full action, whether it be penal torture, purgatorial expiation, or Elysian bliss. Nay, even in the *Iliad*, the opening lines are inexpressibly affecting, where the poet sends the souls of his heroes down to hell, while he leaves their bodies on earth a prey to dogs and all the birds of heaven;* nor less so, where it is said, that the battle shook the earth to its centre, and laid bare the astounded mansions of the dead, in that passage which Longinus extols to the utmost.† There is nothing in literature equally sublime, except in Isaiah, where the dead start from their thrones to hail the fallen monarch of Babylon.‡ But the most startling passage of this kind is that in the *Odyssey*, where the ghost of Achilles tells Ulysses he would willingly return to earth, and labour for ever as a slave and a hireling, rather than remain a prince in the realms beneath.§ Goethe, Byron, and Gray have no imagery similar to this in point of grandeur and solemnity of conception. Perhaps Homer derived his knowledge from some source much nearer to the fountain-head of primeval revelation. It was not till the age of Shakspeare that the Oriental apathy began to manifest itself in this country. It came forward with the dawn of civilization. Hamlet represents the *deadness* of a living soul. Nothing pleased him: everything palled upon his palate: the criminality of his own mother is suggested to him by an act of necromancy, or spiritual intercourse with the nether world; man displeased him much, and woman much more. He loathes his own existence, meditates self-slaughter, kills Polonius, turns Ophelia crazy by his ill-treatment of her, insults those about him, and dies in the midst of bloodshed and shame. Such is one of the most touching and approved dramas of the English stage.

We are almost tempted to imagine that the world has an inherent tendency to paganism—not, indeed, to the worship of

---

* *Iliad*, B. i. 3—5.

† *Iliad*, B. xx. 61. Longin. de Sublim. § ix.

‡ Isaiah xiv. 9.

idols, but to the practice of those principles which were symbolized by idols. There can be no question of the worship of Venus in a way that we need not specify, since no one discredits it; nor is there any doubt of the worship of Bacchus, the god of wine and good cheer; nor of Pluto, the god of wealth. The cultus of these deities is fully recognised in our daily habits, and scrupulously carried out all over Europe. The pagans never paid higher veneration to their deities than we do to ours. The images of these vices are alone wanting to complete their ritual service; and the British nobleman, whoever he was, that facetiously bowed to the statue of Jupiter in the Vatican, was not far off from the truth when he said it was a matter of policy as well of politeness to put himself on good terms with the pagan deities, since no one knew how soon they would come into fashion again. What mean the statues of the great men which adorn our streets? are they not a species of hero-worship? Do they not represent an idea, or a set of ideas, and appeal to those sentiments which mainly actuate the modern world.* Did the idols of antiquity do more or less than this? Cæsar fell at the foot of Pompey's statue, the ideal of conquest; the merchants of Rome celebrated an annual festival to Hermes, the god of commerce; and the statue of *Divus*, or divine Augustus, had, as the superior emblem of power, its appropriate offerings. *Mutato nomine, de te fabula narratur.*

In the first ages of Christianity, it was the opinion of many writers on prophecy, that towards the end of time the world would return to idolatry, actual or potential, and that another Rome would resume the pre-eminence of the first, and surpass her ancient predecessor in wealth, power, splendour, and renown. They supposed that the luxuries of life and the art of living would be carried to the highest pitch; and St. Augustine, in contemplating this surprising course of events, pondered over the loss of Christian faith that was to attend this exalted worldly prosperity.† He asks himself whether it were possible that Christianity should ever perish from off the face of the earth; and yet within three centuries from his death, Mahomet blotted out the name of Christ from a large portion of Asia, the coast of Africa, and the west and east of Europe. Gibbon‡ relates the degradation of the Christians, and says that the light of the

---

* A statue is said to be inaugurated. What is an *inauguration?* The derivative of the word is *augur*, soothsayer. To inaugurate is to consecrate or devote something to a particular purpose. Inauguration, therefore, is an act of devotion, in this particular instance to a statue. Dedicatio magnam religionem habet. *Cicero dedomo suâ.*

† *De Civitate Dei*, lib. xx. c. viii.

‡ *Gibbon*, c. 51, vol. v. 4to ed., p. 386.

Gospel, after a long and perfect establishment, was totally extinguished.*

The foregoing observations have been thrown out for the sake of promoting inquiry, as well as for the purpose of criticising the opinions of an age which excels so greatly in literature, arts, and arms; and it is a fair and legitimate subject of psychological investigation. The habits of thought peculiar to any given period are, if they are wrong, the fault of all or of none. They constitute the prevailing tone of society, and supply the motives of conduct and behaviour, both public and private. They form the atmosphere we breathe, without being aware of its noxious or beneficial effects; and we are taken by surprise when the magic wand of truth dissolves the charm, and shows us the nature of the elements floating around us, their evil and their good, their general bearing on mankind at large, and their specific action on ourselves. It is only by analysing the subject matter of the mind that we discover what the mind itself really is; and the literature of an epoch is the exponent of the progress or decline, the improvement or degeneracy, of a republic, an empire, or the world.

---

